# Analysis of Virion-Incorporated Host Proteins Required for Herpes Simplex Virus Type 1 Infection through a RNA Interference Screen

**DOI:** 10.1371/journal.pone.0053276

**Published:** 2013-01-03

**Authors:** Camille Stegen, Yordanka Yakova, Daniel Henaff, Julien Nadjar, Johanne Duron, Roger Lippé

**Affiliations:** Department of Pathology and Cell Biology, University of Montreal, Montreal, Quebec, Canada; McMaster University, Canada

## Abstract

Viruses are strictly dependent on cells to propagate and many incorporate host proteins in their viral particles, but the significance of this incorporation is poorly understood. Recently, we performed the first comprehensive characterization of the mature herpes simplex virus type 1 (HSV-1) in which up to 49 distinct cellular proteins were identified by mass spectrometry. In the present study, we sought to identify if these cellular factors are relevant for the HSV-1 life cycle. To this end, we performed a small interfering RNA functional screen and found that 15 of these host proteins altered HSV-1 proliferation in cell culture, without any significant effect on cell viability. Moreover, the siRNA used had no negative consequences for Adenovirus type 5 propagation (with one exception) indicating that the modulation was specific for HSV-1 and not merely due to unhealthy cells. The positive host proteins include several Rab GTPases and other intracellular transport components as well as proteins involved in signal transduction, gene regulation and immunity. Remarkably, in most cases when virions were depleted for one of the above proteins, they replicated more poorly in subsequent infections in wild type cells. This highlights for the first time that both the cellular and virion-associated pools of many of these proteins actively contribute to viral propagation. Altogether, these findings underscore the power and biological relevance of combining proteomics and RNA interference to identify novel host-pathogen interactions.

## Introduction

Herpes simplex virus type 1 (HSV-1) virions are composed of a DNA core within an icosahedral capsid surrounded by a heterogeneous and poorly characterized layer of proteins called tegument, which is itself wrapped in an envelope. Many of the tegument components are critical at an early stage of the infection. For example, the binding of incoming viral capsids to microtubules and their transport to the nucleus are dependent on components of the tegument, including the viral proteins UL36 and UL37 [1], [2], [3], [4]. Furthermore, the incoming virion host shut off protein (Vhs; UL41) quickly down regulates the expression of several host proteins following viral entry [5], [6] while VP16, also a tegument protein, regulates the impending cascade of viral gene expression [7]. Interestingly, two other transactivators, namely ICP0 and ICP4, have also been reported in the viral tegument and may play an early role upon entry of the incoming virus [8]. In principle, the incorporation of these molecules should be beneficial to the virus to facilitate the next round of infection.

The importance and complexity of the HSV-1 tegument is illustrated by a recent mass spectrometry study of highly purified extracellular virions, which revealed they contain 23 potential viral teguments and up to 49 distinct cellular proteins [9]. This analysis showed that roughly half of the host proteins found in HSV-1 virions are proteins that had not yet been reported in any herpesviruses. In contrast, the presence of members of the annexin and heat shock protein families as well as cyclophilin A, DDX3X and components of the cytoskeleton have been documented in other *Herpesviridae* [9], [10], [11], [12], [13], [14], [15], [16], [17], [18], [19], [20], suggesting a common function for these proteins. Moreover, host proteins have been documented in numerous other viral particles, including human immunodeficiency virions (HIV [21], [22], [23]), influenza [24], vesicular stomatitis virus [25] and vaccinia [26]. However their biological relevance is, at best, unclear.

The incorporation of host proteins within mature viral particles presumably benefits the virus and may for example jump-start its replication cycle. In contrast, viruses also avoid the incorporation of proteins that can inhibit their replication. One such case is the HIV protein Vif that binds to the host APOBEC3G protein to prevent its inclusion into nascent virions and prevent deamination of the viral genome by this RNA editing protein [21]. It is thus likely that the incorporation of host proteins in viruses is not random but rather a regulated process. Unfortunately, few of these studies have examined the relevance of this phenomenon in the course of an infection [23], [26], [27], [28], [29].

In the present study, we aimed to identify amidst the 49 host proteins found in HSV-1 mature particles those that influence its replication and proliferation. To this end, we designed and validated a functional screening assay using small interfering RNAs (siRNA). We now report that 15 of these proteins have a significant impact on HSV-1 propagation in cell culture, with limited siRNA-associated toxicity or effect on the propagation of another double-stranded DNA virus, the human Adenovirus type 5. Positive hits include proteins involved in vesicular transport, gene regulation, signaling and immunity. Furthermore, we show functional evidence that the incorporation of most of these proteins within mature virions is biologically relevant for HSV-1 infectivity.

## Materials and Methods

### Cells and viruses

143B tk^−^ (ATCC CRL8303) and Vero (ATCC CCL81) cells were grown in Dulbecco's modified Eagle's medium (Sigma-Aldrich) supplemented with 10% fetal calf serum (FCS, HyClone) and 2 mM L-glutamine (Invitrogen) in 5% CO_2_. 143B cells were also supplemented with 15 µg/ml 5-bromo-2 deoxyuridine (BrdU; Sigma) except prior to transfection and infection. The previously described HSV-1 K26GFP mutant (strain KOS) has been provided by Prashant Desai [30]. All viruses were propagated on BHK cells and titrated on Vero cells as previously described. Finally, the pIX-ΔE3 EGFP human Adenovirus type 5 [31] was propagated on 293 cells.

### Antibodies

Primary antibodies were provided and diluted as follows: the anti-DDX3 R648 (1∶1000) was a kind gift from Dr A. Patel [32], while anti-VP16 (1∶1000) and the Remus V anti-HSV-1 antibodies were kindly provided by Dr H. Browne and Dr B. Sodeik respectively. Other antibodies were purchased from commercial sources, including anti-eIF4H (1∶1000, Cell signaling), anti-VP5 (1∶2000; Cedarlane), anti-ICP0 (1∶1000; Abcam), anti-ICP4 (1∶800; Abcam) and anti γ-tubulin (1∶1000; Sigma-Aldrich). Goat anti-mouse and anti-rabbit secondary antibodies were purchased from Jackson ImmunoReasearch.

### Inhibition of HSV-1 life cycle by drugs

143B cells were seeded overnight in a 24-well plate at a 2.5×10^5^ cells/well density. Cells were then mock treated or infected at a multiplicity of infection (MOI) of 5 with HSV-1 K26GFP. After adsorption, cells were washed twice in PBS and fed with complete DMEM containing MG132 (25 µM, Calbiochem), phosphonoacetic acid (PAA; 400 µg/ml; Sigma-Aldrich) or Brefeldin A (BFA; 5 µg/ml; Sigma-Aldrich). In the case of cells treated with MG132, the drug was first added 15 minutes prior to infection and during adsorption in order to prevent the transport of capsids to the nucleus. The infection proceeded for 24 hours at which time supernatants were collected and viral output measured as below (see HSV siRNA screen).

### HSV siRNA screen

siGENOME SMARTpool and select ON-TARGETplus siRNAs targeting the human proteins previously identified in mature extracellular virions [9] were purchased from Dharmacon (Table 1; Thermo Fisher Scientific). The siRNA targeting the HSV-1 protein VP16 was used as a positive control [33] and a scrambled sequence of the VP16 siRNA was used as a negative control. Twenty-four hours prior to transfection, 143B cells were seeded in 24-well plates at a concentration of 5×10^4^ cells/well. siRNA transfections were then performed using Lipofectamine 2000 (Invitrogen) according to the manufacturer's instructions, using 25 or 100 nM/well of siRNA diluted in Opti-MEM or only Opti-MEM as control. The siRNA treated cells were incubated for 5 hours before the medium was replaced with complete DMEM and incubated for 43 more hours for a total of 48 hours of transfection. At that point, cells were mock treated or infected with HSV-1 K26GFP at a MOI of 5. After a one hour adsorption period, cells were washed twice in phosphate-buffered saline (PBS), complete DMEM was added to the wells and the cells were incubated for a further 24 hours. Cells and supernatants were then collected. The viruses in supernatants were inactivated using formaldehyde (Sigma), concentrated 2 hours at 18000× g and the resulting viral pellets resuspended in 100 µl of PBS pH 8. They were then transferred into a 96-well black bottom μClear plate (Greiner Bio-One) and their fluorescence was measured using a Gemini EM spectrofluorometer and SoftMax Pro 4 (Molecular Devices). Alternatively, the infectious virions were titrated on Vero cells as described before [34]. Each experiment was performed in triplicates and repeated three times.

**Table 1:** Proteins tested in the siRNA screen.

Protein Name	Gene ID	Symbol	Catalog Number
14-3-3 protein epsilon	7531	YWHAE	M-017302-02
14-3-3 protein gamma	7532	YWHAG	M-008844-00
14-3-3 protein zeta/delta	7534	YWHAZ	M-003332-01
Alpha actin	58	ACTA1	M-011194-00
Beta actin	60	ACTB	M-003451-03
Gamma actin	71	ACTG1	M-005265-01
Adaptor protein 1 - beta 1 subunit	162	AP1	M-011200-00
Adaptor protein 2 - beta 1 subunit	163	AP2	M-003627-02
Adaptor protein 3 - delta 1 subunit	8943	AP3	M-016014-02
Adaptor protein 4 - epsilon 1 subunit	23431	AP4	M-021474-01
Arf1	375	ARF1	M-011580-01
Arf3	377	ARF3	M-011581-00
Arf4	378	ARF4	M-011582-01
Arf5	381	ARF5	M-011584-01
ATP dependant RNA helicase DDX3X	1654	DDX3X	M-006874-01
Casein kinase 2	1457	CSNK2A1	M-003475-03
Cofilin 1	1072	CFL1	M-012707-00
Cyclophilin A	5478	PPIA	M-004979-01
Cystein-glycin rich protein 1	1465	CSRP1	M-011839-02
Eukaryotic translation initiation factor 4H	7458	EIF4H	M-013054-00
Golgi-associated, gamma adaptin ear containing, ARF binding protein 1	26088	GGA1	M-013694-01
Golgi-associated, gamma adaptin ear containing, ARF binding protein 2	23062	GGA2	M-012908-01
Golgi-associated, gamma adaptin ear containing, ARF binding protein 3	23163	GGA3	M-012881-00
Glyceraldehyde-3-phosphate dehydrogenase	2597	GAPDH	M-004253-02
Growth factor receptor bound protein 2	2885	GRB2	M-019220-00
Heat Shock 70 protein 8^a^	3312	HSPA8	M-017609-01
Keratin 1	3848	KRT1	M-012865-02
Keratin 10	3858	KRT10	M-023057-00
Macrophage Migration Inhibitory Factor	4282	MIF	M-011335-01
CD59^b^	966	CD59	M-004537-01
NM23A^c^	4830	NME1	M-006821-01
NM23B^c^	4831	NME2	M-005102-02
Peroxiredoxin-1	5052	PRDX1	M-010338-00
Peroxiredoxin-2	7001	PRDX2	M-008178-03
Profilin-1	5216	PFN1	M-012003-01
Programmed cell death protein 6	10016	PDCD6	M-004440-03
Rab2A	5862	RAB2A	M-010533-01
Rab2B	84932	RAB2B	M-010370-00
Rab4B	53916	RAB4B	M-008780-03
Rab5A	5868	RAB5A	M-004009-00
Rab5B	5869	RAB5B	M-004010-01
Rab5C	5878	RAB5C	M-004011-01
Rab6A	5870	RAB6A	M-008975-01
Rab6B	51560	RAB6B	M-008548-02
Rab6C	84084	RAB6C	M-009031-02
Rab7A	7879	RAB7	M-010388-00
Rab10	10890	RAB10	M-010823-01
Rab11A	8766	RAB11A	M-004726-02
Rab11B	9230	RAB11B	M-004727-02
Rab15	376267	RAB15	M-031564-00
Rab33B	83452	RAB33B	M-008909-02
Rab35	11021	RAB35	M-009781-00
Rab-like protein 3	285282	RABL3	M-018128-01
S100 calcium protein binding A11	6282	S100A11	M-012138-00
SEC14-like 4	284904	SEC14L4	M-019246-00
Tetraspanin 13	27075	TSPAN13	M-012516-01
Transferrin Receptor (CD71)	7037	TFRC	M-003941-02
Transgelin 2	8407	TAGLN2	M-011468-02
Translocase of inner mitochondrial membrane 50 homolog	92609	TIMM50	M-023692-01
Triosephosphatase isomerase	7167	TPI1	M-009776-02
Ubiquitin C	7316	UBC	M-019408-01
Ubiquitin-conjugating enzyme E2L 3	7332	UBE2L3	M-010384-01

a HSP70 (Loret, 2008).

b Membrane attack complex inhibition factor (Loret, 2008).

c Nucleoside diphosphate kinase A/B (Loret, 2008).

### Adenovirus siRNA screen

143B cells transfected for 48 hours with Lipofectamine alone or 25 nM siRNA as described above were mock treated or infected with the Adenovirus pIX-ΔE3 EGFP at an equal MOI for 48 hours. Supernatant were collected and viral DNA was purified using the GenElute Mammalian Genomic DNA Miniprep Kit (Sigma-Aldrich) according to the manufacturer's instructions. qPCR was performed with a Rotor-Gene 6000 (Corbett) using the FastStart SYBR Green Master (Roche Diagnostics). For the amplification, the primers pairs specific for EGFP were used: forward, 5′ ACG TAA ACG GCC ACA AGT TC 3′; reverse, 5′ AAG TCG TGC TGC TTC ATG TG 3′. The data are expressed in relative genome copy compared to the Lipofectamine-treated control and were pooled from three independent experiments. For these experiments, siRNA targeting VP16 served as negative control whereas a siRNA targeting the Adenoviral hexon protein [35] was used as positive control.

### Viability assays

143B cells were mock treated with Lipofectamine alone or transfected with siRNA for 44 hours. Cells were washed twice and the medium replaced. alamarBlue (AbD Serotec) was subsequently added to the cells or to DMEM medium as a control according to the manufacturer's instruction. The cells were further incubated in accordance with the manufacturers' instructions before viability was measured by spectrofluorometry.

### Two-steps infection assays

143B cells were transfected for 48 hours as in the siRNA screen or with Lipofectamine only. Cells were then infected for a further 24 hours with HSV-1 K26GFP at a MOI of 5. Supernatants, containing viruses depleted of individual host proteins, were collected and titrated on Vero cells. Fresh 143B cells were either mock treated or transfected with the siRNA for 48 hours. They were then infected with the depleted virions at an MOI of 0.1 for 48 hours. The supernatants form this second round of transfection/infection were collected and titrated once again on Vero cells.

### Western blotting

43B cells were transfected for 48 hours with Lipofectamine alone or siRNAs then were mock treated or infected with K26GFP at a MOI of 5 for 24 hours. After a centrifugation of 5 min at 1000×g, the cells were collected, washed in PBS and lysed by 3 cycles of rapid freeze-thaw. Infected cell's supernatants were concentrated for 2 hours at 18 000× g and resuspended in 10 µl of PBS. Unless specified otherwise, equal amount of proteins were analyzed by SDS-PAGE [9] [12]. Antibodies against the viral proteins VP5 and VP16 or the human DDX3X, ARF1, eIF4H and γ-tubulin proteins were used to blot the respective proteins. When indicated, protein expression levels were quantified using ImageJ (version 1.45b).

### Statistical analysis

Fluorescence and virus titers were normalized to the values obtained for the controls as indicated in the figure legends. Bilateral Student T tests were performed using GraphPad Prism 5 (GraphPad Software).

## Results

### Validation of the siRNA-based assay

**Figure 1:**
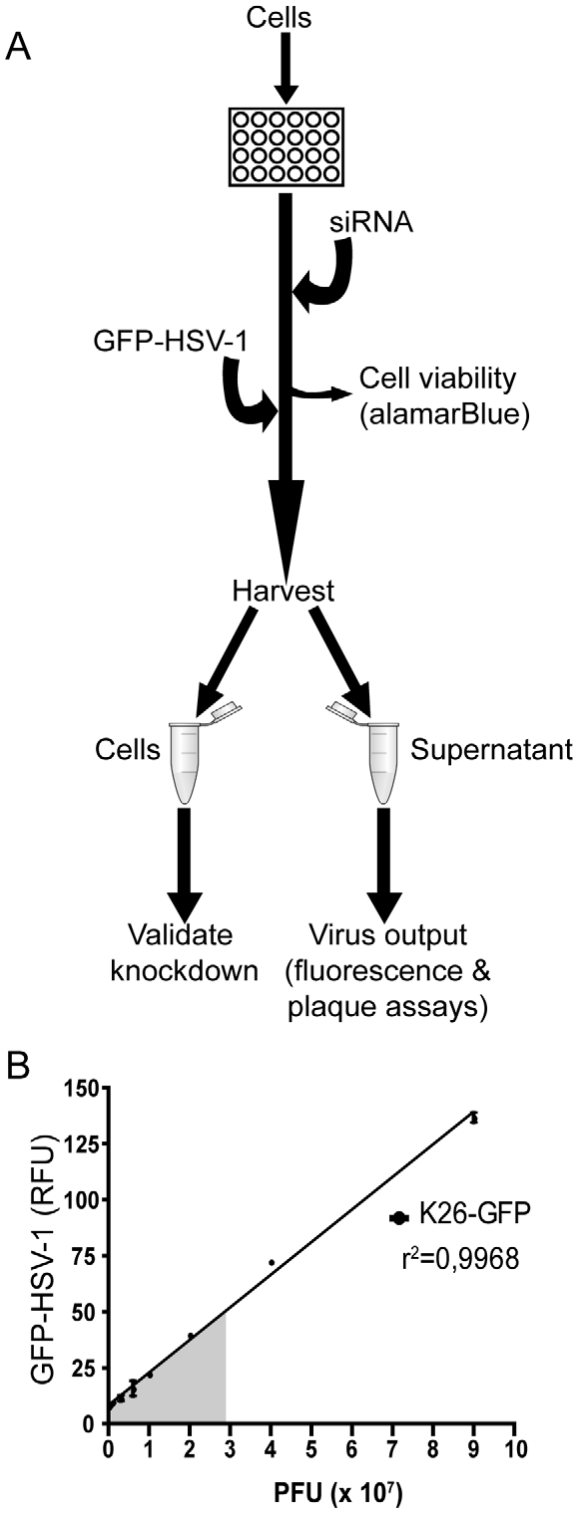
Screening method. **A**) 143B cells were seeded in 24-well plates 24 hours prior to transfection. Cells were then transfected with siRNA and incubated 48 hours before being infected with HSV-1 K26GFP for an additional 24 hours. Supernatants were collected and their fluorescence was measured using a Gemini EM spectrofluorometer. As a cytotoxicity control, cell viability was assessed in parallel using alamarBlue 2 days post-transfection. In addition, the cells were lysed and used for Westerns to validate the siRNA knockdowns. **B**) The fluorescence of pre-titered infectious HSV-1 K26GFP particles serially diluted in PBS was quantified using a spectrofluorometer. The fluorescence obtained was linear with the titers through the entire selection (r^2^ = 0.9968), thus demonstrating the advanced sensitivity of the device. The gray area denotes the typical of values obtained in the RNA interference screen below. RFU: relative fluorescence units.

Our strategy to first examine the physiological relevance of the host proteins identified in mature HSV-1 virions involved a novel viral propagation assay based on RNA interference and HSV-1 K26GFP, a wild-type virus in which the *Aequorea victoria* green fluorescent protein (GFP) is fused to the capsid protein VP26 [30] (Figure 1A). This approach enabled us to easily and rapidly measure viral output and to quantitatively screen many targets without resorting to the classical but time-consuming and cumbersome plaque assays. We selected a human cell line for this screen because it is the HSV-1 natural reservoir, it is compatible with our previous proteomics report [9] and a human siRNA library is commercially available. We opted for the human osteosarcoma-derived 143B cell line since it is more resistant to the cytopathic effects of the virus and produces significantly greater quantities of extracellular viruses upon infection than the HeLa cells originally used in our proteomic study ([34], [36] and data not shown). In addition, 143B cells have a greater than 80% siRNA transfection rate (data not shown). Cell plating density, infection conditions, harvesting time, assay buffers, plate format and parameters of the plate reader software were all extensively optimized (data not shown) to ensure that quantification of the virus from the supernatant was accurate, linear and sufficiently sensitive to detect extracellular virions (Figure 1B).

**Figure 2:**
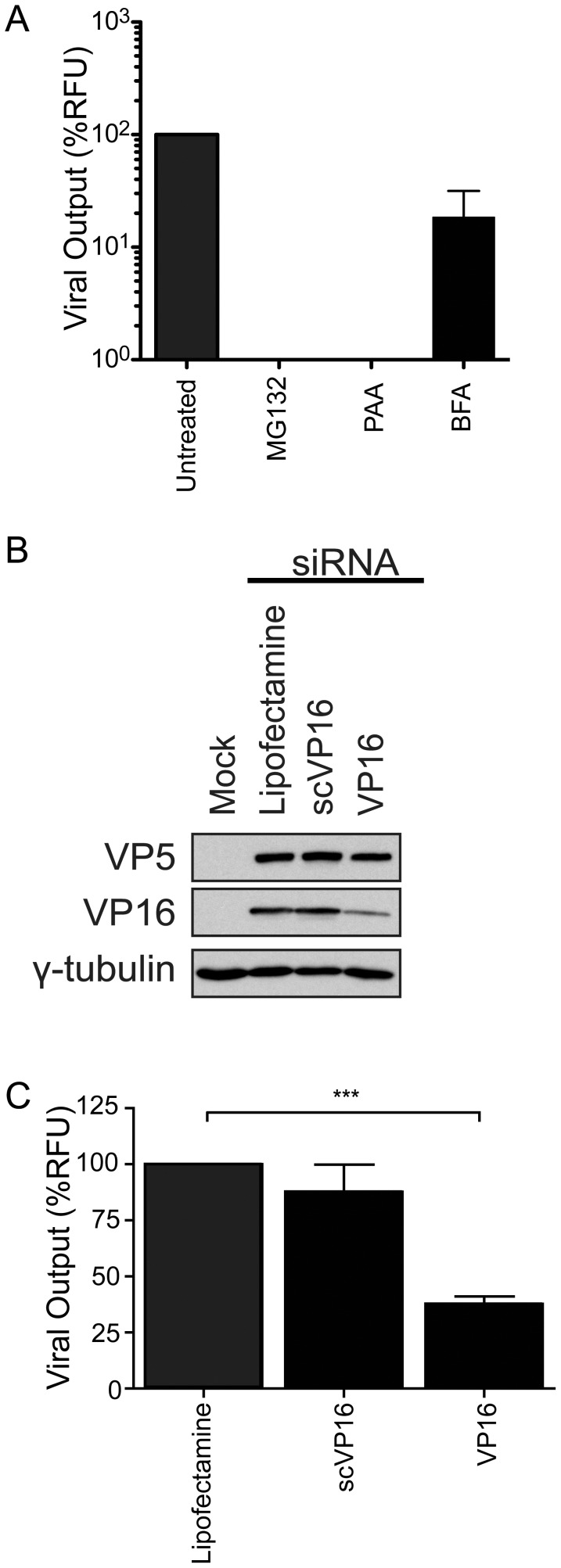
Validation of the assay. 143B cells were seeded in 24-well plates and infected with HSV-1 K26GFP and **A**) treated with drugs targeting HSV-1 post-entry (MG132), replication (PAA) and egress (BFA). **B–C**) As above except that cells were instead transfected for 48 hours prior to infection with Lipofectamine only, a scrambled version of the VP16 siRNA or siRNA targeting VP16. For panel A and C, the fluorescent viruses in the supernatants were concentrated by centrifugation at 24 hpi and quantified by spectrofluorometry. For panel B, 25 µg of proteins from cell lysates were tested by Western blotting for VP16 knockdown. γ-tubulin was used as loading control. The error bars show the standard errors of the mean (SEM) of three independent experiments. Bilateral Student's T-tests were performed to detect significant hits compared to the siRNA free control (***: p<0.0001).

We next sought to validate that the assay could indeed detect the impact of known inhibitors of the HSV-1 life cycle. We therefore pretreated cells with MG132, an inhibitor of the proteasome that perturbs the post-entry delivery of HSV-1 to the nucleus [37], phosphonoacetic acid (PAA) which prevents viral replication [38] and brefeldin A (BFA) which arrests viral egress of newly synthesized viral particles [39], [40]. As expected, HSV-1 output was drastically lower in drug-treated cells than in untreated ones (Figure 2A). As a second control, cells were transfected with siRNA targeting the HSV-1 protein VP16 (UL48), since its inhibition by siRNA is known to efficiently reduce VP16 expression and viral production [33]. Cells were thus transfected for 48 hours prior to infection with the single most effective siRNA targeting VP16 [33] or with Lipofectamine only. A scrambled sequence of the VP16 siRNA was used as negative control (scVP16) Since the scVP16 siRNA does not have any homology to any human or viral sequence as determined by blast (data not shown), it also served as a non-targeting control. The knockdown of VP16 expression was assessed by Western blotting and quantified with ImageJ, which revealed the high efficacy of the siRNA employed (up to 88% inhibition; Figure 2B). Though the inhibition of VP16 was not absolute, the extracellular viral yields were significantly lower upon treatment with siRNA against VP16 than for cells treated with the transfection agent only or the scrambled control (Figure 2C). Taken together these results demonstrate the sensitivity and specificity of the assay.

### Assessment of host proteins critical for the viral infection

**Figure 3:**
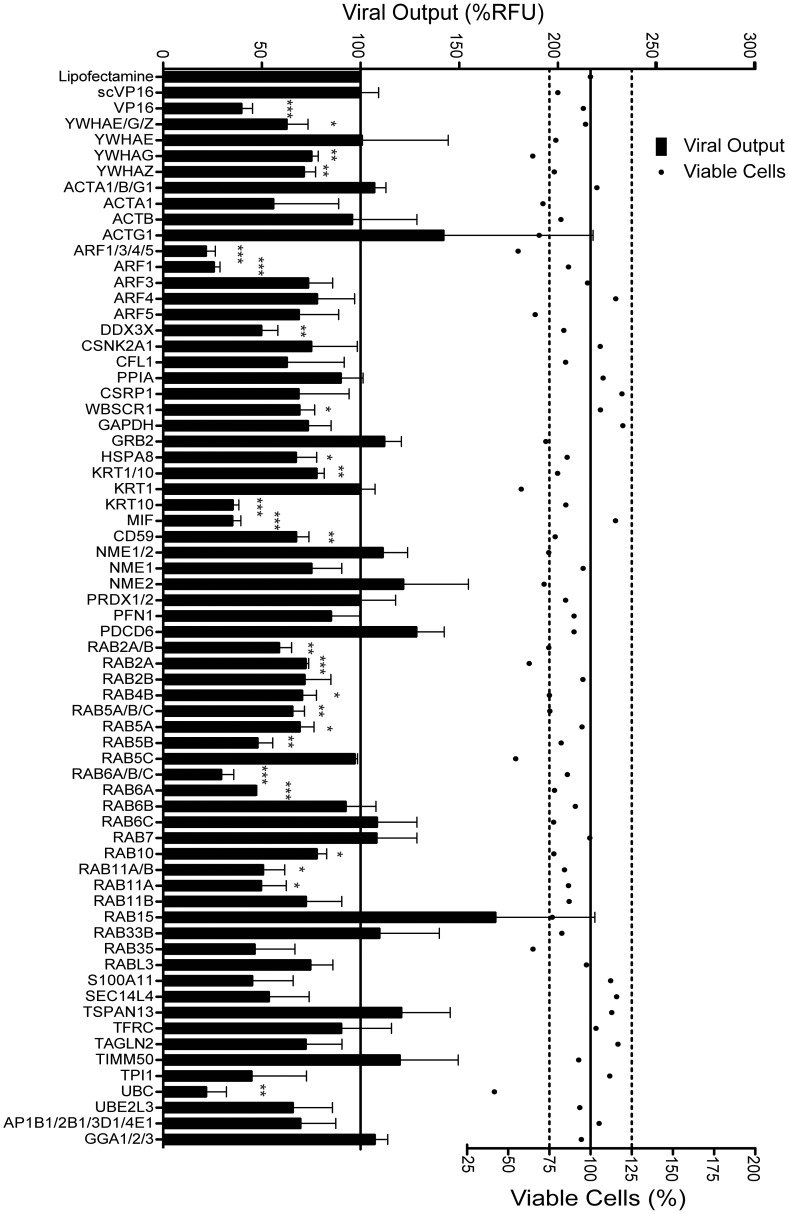
siRNA screen against the host proteins identified in mature extracellular HSV-1 virions. 143B cells were transfected for 48 hours with Lipofectamine alone or with siRNA duplexes targeting the indicated cellular proteins (gene names are indicated on the left). Forty-eight hours later, cellular viability was assessed by alamarBlue or cells were infected in parallel with K26GFP at a MOI of 5 for a further 24 hours. The fluorescence in the supernatant was quantified using a spectrofluorometer. The data was normalized to the mean value obtained with Lipofectamine only samples. Bilateral Student's T-tests were performed to detect significant hits compared to the Lipofectamine only control (*: p<0.05, **: p<0.001, ***: p<0.0001). The solid line represents the relative fluorescence intensity of the Lipofectamine treated samples while the dotted lines indicate ±25% viability. The error bars show the standard errors of the means (SEM) of three independent experiments, each done in triplicates.

To evaluate the functional relevance of the cellular proteins identified in mature extracellular virions, we examined them using the above siRNA screen. Since some of the peptides that were identified in the course of the proteomic study are common to different protein isoforms, we screened them together but also individually, thus lengthening our original list of proteins to 55 (Table 1). Furthermore, we added two additional specificity controls that were not in our initial proteomics study. They are the adaptor proteins (AP1 to AP4) and GGA proteins (1 to 3), all molecules regulating protein transport and relevant in the view of the many transport molecules reported here. The siRNA targeting VP16 and its scrambled version (scVP16) were once again used as controls. For each of the human targets identified in our proteomic study [9], we used a pool of four different siRNA duplexes to ensure the most effective knockdowns. These siRNA pools are commercially available and predefined by the manufacturer according to their proprietary algorithm (see Table 1 for catalogue numbers). Cells were transfected in triplicates with these siRNA for 48 hours, to allow endogenous levels of the host proteins to significantly decrease, and subsequently infected with GFP tagged HSV-1. Twenty-four hours later, viral yields were measured. Though both the intracellular and extracellular viral pools could be quantified, we opted to only probe the latter since free VP26-GFP is inevitably present in infected cells and would contribute to a higher background. Each experiment was normalized to the mean fluorescence obtained in supernatants from cells transfected with the transfection agent only and results from three independent screens were compiled. This method allowed us to identify several cellular proteins whose absence statistically altered viral production (p<0.05 or better; Figure 3). To evaluate the toxicity of the siRNA reagents and rule out nonspecific effects, the viability of the cells was monitored in parallel using the redox indicator alamarBlue, which quantitatively monitors cell metabolism and, consequently, overall cell viability (Figure 3, dots). Among the proteins that affected viral output, only the inhibition of ubiquitin C resulted in high cytotoxicity under our experimental conditions and was not pursued further. It is relevant to note that targeting some of the proteins with siRNA resulted in lower cellular viability without any impact on viral production (e.g. KRT1 and RAB5C), further highlighting the genuine implication of the other cellular proteins on the HSV-1 viral life cycle.

**Figure 4:**
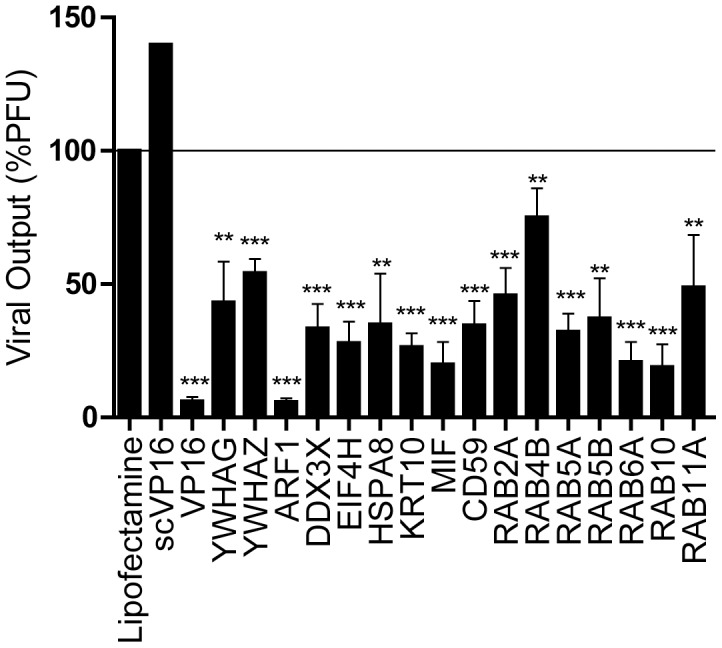
Impact of host protein depletion on infectious particles. 143B cells were transfected with siRNA and infected with K26GFP as before for the samples that were positive hits in Figure 3. Supernatants were individually collected and their viral content titrated on Vero cells. The titers were normalized to the mean value obtained with samples treated with Lipofectamine only. The error bars show the standard errors of the means (SEM) of three independent experiments, each done in duplicates. Bilateral Student's T-tests were performed to detect significant hits compared to the Lipofectamine only control (**: p<0.001, ***: p<0.0001). PFU: Plaque forming units.

Given that the GFP-based assay used in this study detects total viral particles rather than infectious virions, single positive isoforms were confirmed by conventional plaque assays to measure viral titers following siRNA treatment. 143B cells were transfected with siRNA, infected as before and the infectious particles in the supernatant were titrated on Vero cells. As expected, infected cells treated with the scrambled control produced similar levels of extracellular infectious particles as the Lipofectamine-treated sample. Moreover, all the siRNA tested lead to a statistically significant reduction in viral yields and were thus fully coherent with the GFP detection assay (Figure 4). Since Rab4B inhibition reduced viral yields by less than 25%, it was therefore excluded from further analysis. Taken together, these data validated the GFP-based readouts and show that the individual depletion of host proteins incorporated in mature HSV-1 virions impacts both total and infectious particle yields.

**Figure 5:**
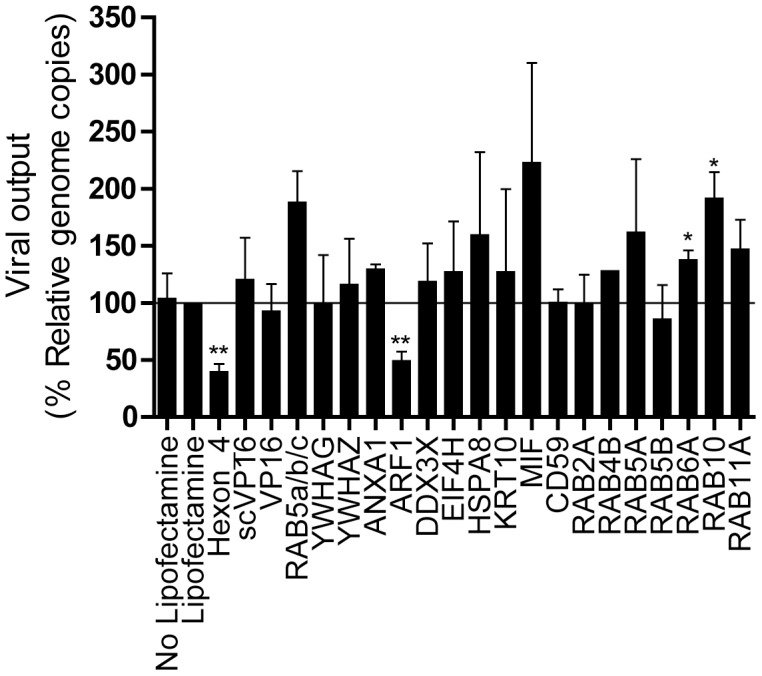
Impact of the HSV-1 virion-incorporated cellular proteins on the life cycle of Adenoviruses. 143B cells were transfected with siRNA and infected with HAd pIX ΔE3 GFP as in materials and methods for the samples that were positive hits in Figure 3. Supernatants were individually collected and their viral content titrated by qPCR. The titers were normalized to the mean value obtained with samples treated with Lipofectamine only. The error bars show SEM of three independent experiments. Bilateral Student's T-tests were performed to detect significant hits compared to the Lipofectamine only control (*: p<0.05, **: p<0.001).

Thus far, our data support the potential involvement of several host proteins in the HSV-1 life cycle. To evaluate if these cellular proteins specifically affect HSV-1 propagation rather than non-specifically perturb cell metabolism, 143B cells were transfected with siRNAs targeting the positive hits and infected with human Adenovirus type 5, another double-stranded DNA virus that replicates in the nucleus. In this experiment, the siRNA targeting the HSV-1 protein VP16 served as negative control whereas a siRNA targeting the hexon protein [35] was used as positive control. As expected, siRNA targeting the adenoviral hexon reduced viral yields (68% inhibition) while siRNA targeting VP16 did not (Figure 5). Interestingly, only Arf1, Rab6A and Rab10 statistically influenced Adenovirus proliferation. This strongly suggests that the positive hits found in our screen specifically modulated HSV-1 proliferation and supports the conclusion that the effects observed were not due to some general cellular defects.

### Importance of the proteins present in the mature virions

Several viruses have been reported to incorporate cellular proteins but the significance of this incorporation is often unclear. There are various reasons why cellular proteins could be incorporated in virions. Some of them may simply be incorporated in virions because they are abundant and/or happen to be at the right place and time but are not valuable to the virus. A second possibility is they may be included as a consequence of their function during viral assembly, intracellular transport or egress. In such cases, the proteins would not necessarily be important to initiate the next round of infection. Finally, some proteins may specifically be required to promote the infection of neighboring cells. In this exciting scenario, the depletion of the cellular proteins from the viral particles should stop or delay the next replication cycle, much the same way VP16 is incorporated into mature virions to act early during the infection as transactivator of immediate early genes [7]. We thus wished to directly address the functional significance of the virion-associated host proteins.

**Figure 6:**
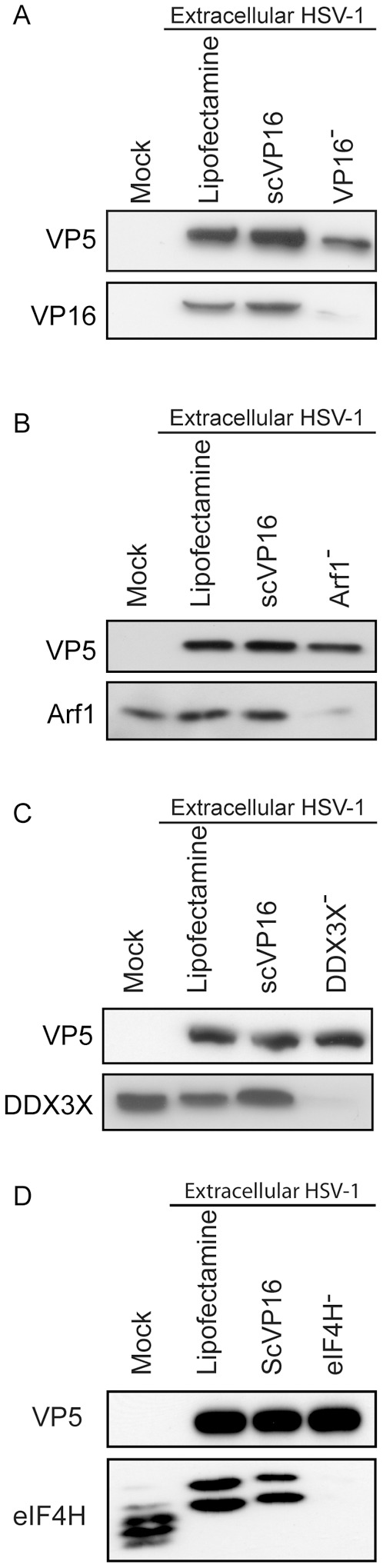
Virions harvested from siRNA treated cells are efficiently depleted of the siRNA-targeted protein. 143B cells were transfected with Lipofectamine only or siRNAs against the control VP16 or the positive hits Arf1, DDX3X and eIF4H. They were then infected at a MOI of 5 with HSV-1 K26GFP and the supernatants collected at 24 hours. Concentrated supernatants corresponding to equal amounts of VP5 were analyzed and 20 µg of Lipofectamine-treated cell lysates were used as control. The reduced levels of **A**) VP16, **B**) ARF1, **C**) DDX3X and **D**) eIF4H in newly produced virions in the supernatants were confirmed by Western blotting.

**Figure 7:**
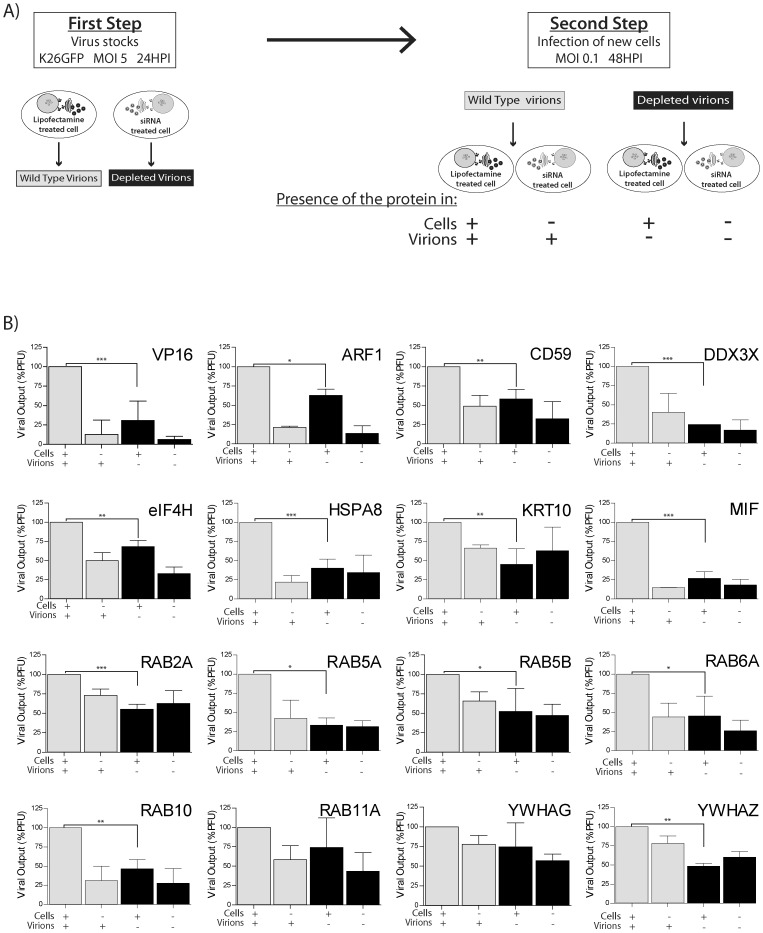
Importance of the virion-incorporated pools of the proteins. **A**) As described in materials and methods, a two step approach was used. In the first step, virions were harvested from 143B cells individually treated with siRNA against 15 cellular proteins (DDX3X, MIF, RAB5A, HSPA8, KRT10, RAB6A, RAB10, YWHAZ, RAB5B, RAB2A, CD59, YWHAG, ARF1, eIF4H and RAB11A) and infected with wild type HSV-1 K26GFP at a MOI of 5 for 24 hpi. As control, viruses produced on Lipofectamined-treated cells were also produced. The extracellular virions were collected, titrated and used to infect a fresh layer of 143B cells transfected or not with siRNA at an MOI of 0.1 for 48 hours (**second Step**). The presence of the cellular proteins in the virus or in the cells is indicated with +/− under each condition of infection. **B**) The supernatants form this second infections were collected and titrated on Vero cells. Viral titers were normalized to the mean value of Lipofectamine-treated cells infected with wild type virus (+/+). The presence or absence of the cellular proteins in the cell, in the virus or in both is presented with + and − under each graphic. To statistically evaluate if the viral pool of the host proteins modulate the propagation of HSV-1, the depleted virions (3^rd^ bar) were compared to the Lipofectamine only control (1^st^ bar) using bilateral Student's T-tests (*: p<0.05, **: p<0.001, ***: p<0.0001).

Since the virion-incorporated host proteins are present in both mature virions and the cell, the respective contribution of both of these pools to the viral life cycle was assessed. To this end, a two-step approach was employed. In the first step, viruses individually depleted for each of the positive targets identified above were produced on siRNA-treated cells. As controls, we also produced virions on Lipofectamine-treated cells or cells treated with VP16 or scVP16 siRNAs. The efficacy of the depletion was then examined by Western blotting for four of the proteins. As shown in figure 6, the siRNA strongly reduced the amount of the proteins in extracellular virions . It was thus possible to produce stocks of virions depleted on a given host protein. These viruses were then titrated and used to infect a new monolayer of cells (second step; Figure 7A). To reduce the risk of complementation between depleted virions and wild type virions produced by cells that may not have taken up the siRNA, a low MOI of 0.1 was employed. To sort out the importance of the viral and cellular pools of the proteins, all four combinations of wild type/depleted virions and wild type/depleted cells were probed. As expected, the infection of VP16 siRNA-treated cells with wild type virus harvested from Lipofectamine-treated cells strongly reduced viral yields, in accordance with the idea that newly expressed VP16 does regulate the viral life cycle (Figure 7B). Though not as extensive, the infection of Lipofectamine-treated cells with VP16-depleted virions also significantly reduced viral production, consistent with past reports that the virion associated pool of VP16 is actively involved in the early stages of the infection process [7]. Moreover, the depletion of both newly expressed and virion-associated VP16 (i.e. infection of VP16 siRNA-treated cells with VP16-depleted virions) further reduced viral output compared to the virion-only depleted sample, hence reiterating the importance of both newly expressed and virion-associated VP16 pools during the HSV-1 infection. Interestingly, the individual depletion from the mature virions of 13 of the 15 target tested statistically reduced viral production in Lipofectamine-treated cells, 8 of which did so by at least 50% (DDX3X, HSPA8, KRT10, MIF, RAB5A, RAB6A, RAB10 and YWHAZ (Figure 7B). One notable case was Arf1, for which the cellular pool clearly participated in greater proportion to viral propagation, despite the significant contribution of the viral pool of this protein. Given the above findings occurred in the presence of a normal complement of host proteins, this reemphasized the specific involvement of these proteins in HSV-1 replication (Figures 3 and 4) rather than an indirect metabolic defect. Altogether, this directly demonstrates for the first time that both the cellular and the virion-incorporated pools of these proteins do indeed participate in the HSV-1 replication cycle.

## Discussion

Viruses are obligatory intracellular parasites that depend on many cellular functions to complete their life cycle. These interactions are complex and often poorly understood. While the contribution of host proteins to HSV-1 has been partially elucidated, genomic screens with other viruses suggest this may only be the tip of the iceberg [41], [42], [43], [44], [45]. Recently, we reported the detection of up to 49 distinct cellular proteins within mature extracellular HSV-1 virions [9], adding an additional layer of complexity to this scenario. Since some of these proteins may play pivotal roles during the viral life cycle, they constitute prime targets to identify and characterize novel host-pathogen interactions in the context of an HSV-1 infection.

The siRNA assay reported in this study is sensitive, rapid, linear and correlates with classical plaque assays (Figures 1 to 4). The data obtained by both fluorometry and plaque assays suggest that at least 15 of the cellular proteins tested are involved in the HSV-1 replication cycle in cell culture (Figures 3 and 4). Moreover, the incorporation into mature extracellular virions of most of these proteins seems necessary to ensure an optimal new round of infection (Figure 7). It should be noted that overexpression of several of these proteins did not influence viral yields, presumably because they are not rate limiting in the cell (data not shown). These results are unlikely due to side effects of the depletion of the host proteins within the cells or to off-targeting effects of the siRNA. Indeed, first of all, only limited cytotoxicity was noted under our experimental conditions, which is consistent with other genomic screens [44], [46]. Secondly, even when siRNA were somewhat toxic (e.g. KRT1 and Rab5C), this did not cause a reduction of viral yields. Only the inhibition of ubiquitin C, which did perturb HSV-1 output, correlated with high siRNA-associated cytotoxicity and was therefore not considered further, though it may still be relevant for the HSV-1 replication cycle (Figure 3). Third, we could single out specific protein isoforms that affect viral proliferation, while other isoforms of the same proteins had no impact. For instance, inhibition of Arf1 significantly reduced viral production while the inhibition of Arf3, 4 or 5 failed to do so, hinting at the specificity of the assay and the lack of pleiotropic effect of the siRNAs (Figure 3). Forth, the same siRNA, with the sole exception of Arf1, Rab6A and Rab10, did not significantly impact the replication of Adenovirus type 5 (Figure 5). In that case, it remains to be seen if these proteins are essential to viral growth under our experimental conditions or if both HSV-1 and Ad5 propagation depends on these host proteins. Finally, depleting these host proteins from the virions, but not the cell, still had an impact on viral yields (Figure 7). We therefore conclude that these molecules are most likely involved in the viral life cycle and that our observations cannot be credited to some pleiotropic effect.

It is important to note that the actual number of proteins regulating the HSV life cycle in the original RNA interference screen is likely higher than reported in the present study. Missed targets could include cellular proteins with long half-lives unperturbed by the siRNA, proteins with more subtle impacts on the virus as well as proteins that may be complemented by their virion-associated counterpart or by other cellular proteins. Consistent with this latter scenario, it is worth noting that depletion of either the cellular and virion pools of the proteins often affected viral propagation. Furthermore, the screen only considers host proteins involved in HSV-1 replication in tissue culture and may exclude some virulence factors and immune or latency-related proteins. Finally, the screen initially measured GFP output and quantified total viral particle release rather than infectious virions as determined by plaque assays. It is thus possible that some siRNA reduced the production of infectious viruses without affecting the total amount of viral particles, as noted when VP16 is inactivated [47]. Despite these limitations, the positive rate from this study is very high at 31% (i.e. 15/49). For comparison, genome-wide siRNA screens for HIV, influenza and West Nile virus have positive rates below 1.5% [41], [42], [43], [44], [45], [48]. The data thus constitute a strong validation of the relevance of our strategy to unmask novel cellular players by combining proteomics [9] and a targeted siRNA screen (this study). Furthermore, this study and those of others [5], [33], [49], [50], [51], [52] highlight the potency of siRNA as a useful tool to study HSV-1 host-pathogen interactions, despite of the ability of the virus to downregulate RNA-induced gene silencing [53].

The current GFP-based assay is less sensitive than traditional plaque assays but is a good complementary approach to screen large numbers of targets. While it is clear that the reductions are not as spectacular as when some viral genes are deleted, important differences have to be considered when analyzing the impact of host proteins instead of viral proteins. For instance, host proteins are often complemented by another gene copy, another isoform, a parallel pathway or other means, which implies that smaller effects upon depletion should not be surprising. This is even true for some HSV-1 genes, for which multiple deletions are required to see a significant phenotype. Moreover, cellular proteins finely exert their actions with impacts typically in the lower fold range (2–4 folds) rather than logs (10–100 folds). In agreement with this view, a study by Hill and colleagues [54] showed that HSV-1 itself modulates the expression of 3,123 host proteins by only 1.4 to 14.2 folds, changes that are presumably beneficial for the virus. Our results are also in the same range (i.e. less than a log difference) observed by others in recent studies analyzing the impact of cellular proteins on HSV or HIV proliferation [55], [56], [57], [58], [59], [60], [61]. Multi-log inhibitions where therefore not expected in the present study but it does naturally make it more difficult to ascribe a functional role to present host proteins. However, the significant, albeit moderate, impact of depleting the host proteins in the virions themselves, while leaving the cellular pools intact, strongly supports their contribution to the HSV-1 replication cycle. The present data suggest that many of the tested proteins are indeed subtle modulators of viral production.

**Figure 8:**
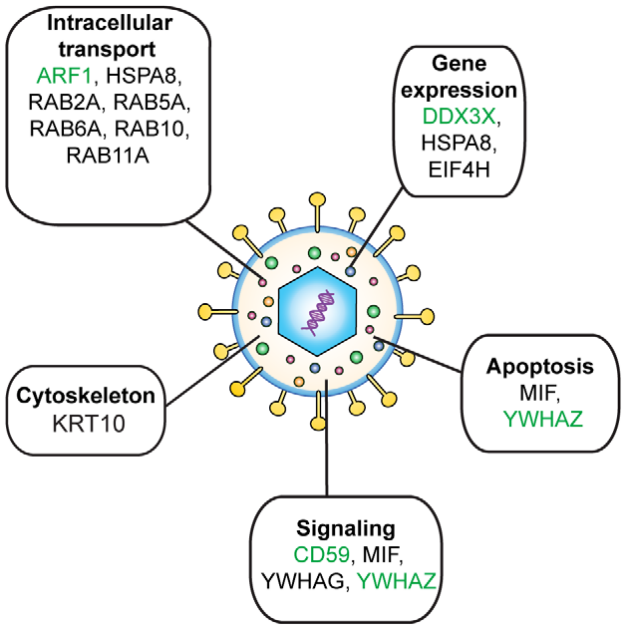
Cellular functions of virion-incorporated host proteins. Several of the positive hits of our functional screen are implicated in pathways that are most likely relevant for the virus. While all of them were found in HSV-1 mature viral particles, many of these proteins (in green) have also been found incorporated into other herpesviruses, suggesting a common role for these proteins throughout the family [12], [13], [14], [17].

The positive hits found in this study identified several potential players in the HSV-1 life cycle. Many of these proteins are involved in pathways that are most likely used in the infection process (Figure 8). Among them, several Rab GTPases and other intracellular transport components as well as proteins involved in signal transduction were identified, some of which have already been proven necessary for viral production. For instance, it has recently been shown that Rab6A is necessary for the cellular trafficking of HCMV viral protein pp150 and efficient virus assembly [57], while Rab11 modulates the intracellular transport of influenza genome to the plasma membrane [62]. Similarly, Rab1 and Rab43 have been implicated in HSV-1 secondary envelopment [55]. It is thus highly possible that some of the proteins identified in the present study are involved in the numerous transport events occurring in the course of HSV-1 infection. The present screen is therefore very useful to pursue novel host-pathogen interactions in the context of HSV-1 infections. Moreover, 6 of the proteins; 14-3-3 ζ/δ (YWHAZ), Arf1 (ARF1), DDX3X, eIF4H, keratin 10 (KRT10) and CD59 have previously been found in HCMV, KSHV and/or PRV viral particles in addition to HSV-1 [10], [12], [13], [14], [17], suggesting that they might have a common roles throughout the herpesvirus family.

Many studies have reported the incorporation of host proteins within mature virions but very few have addressed the significance of the incorporated fraction of cellular proteins in viral infectivity. To verify the importance of the pool of host proteins present in the extracellular virion, we depleted these proteins from virions by RNA interference and used them to re-infect untreated cells. This method enabled us to identify for the first time 8 proteins whose absence from the virions reduced viral production by at least 50%, with evidence of contributions by another 5 host proteins (Figure 7). This highlights the very close relationship between the virus and its host. In contrast, the depletion of some host proteins (i.e. Rab11A and YWHAG) in the virions caused no significant impact on viral production. It may be relevant to highlight that the present study was performed in 143B cells and it is likely that the pool of host proteins incorporated in mature virions or their roles varies among cell types. Though difficult to perform, a comprehensive study of host proteins in virions produced on neuronal cells would be particularly interesting.

The present study highlights the very close relationship between HSV-1 and its host. However, the full extent of host-pathogen interactions between HSV-1 and the cell remains elusive at the moment. Our approach constitutes an innovative and powerful strategy to identify potential novel cellular proteins that modulate the HSV-1 life cycle. The characterization of their functions at the molecular level is therefore of the upmost interest. Since all of these proteins have previously been detected in mature extracellular HSV-1 virions [9], they constitute a new class of proteins to study as potential therapeutic targets.
